# Circadian Clock Dysregulation and Prostate Cancer: A Molecular and Clinical Overview

**DOI:** 10.1177/11795549231211521

**Published:** 2023-11-27

**Authors:** Dalia Kaakour, Bridget Fortin, Selma Masri, Arash Rezazadeh

**Affiliations:** 1 Division of Hematology/Oncology, Department of Medicine, University of California, Irvine, Orange, CA, USA; 2 Department of Biological Chemistry, University of California, Irvine, Irvine, CA, USA

**Keywords:** Prostate cancer, circadian rhythm, clock dysregulation, oncogenesis, shift work

## Abstract

Circadian clock dysregulation has been implicated in various types of cancer and represents an area of growing research. However, the role of the circadian clock in prostate cancer has been relatively unexplored. This literature review will highlight the potential role of circadian clock dysregulation in prostate cancer by examining molecular, epidemiologic, and clinical data. The influence of melatonin, light, night shift work, chronotherapy, and androgen independence are discussed as they relate to the existing literature on their role in prostate cancer.

## Introduction

Prostate cancer has an estimated national incidence of 268 490 cases in 2022.^1^ Approximately 1 in 8 men living in the United States will be diagnosed with prostate cancer during their lifetime.^2^ Research into the mechanistic underpinnings driving prostate cancer development and progression has focused predominantly on androgen receptor–dependent mechanisms. There is growing yet insufficient evidence regarding androgen receptor–independent mechanisms for progression of prostate cancer, including the impact on nutrition, genomics, and inflammation. Recent evidence suggests a role for the circadian clock in prostate cancer development, highlighting the need for a better understanding of how dysregulated circadian rhythms contribute to tumorigenesis. This review will provide a comprehensive overview of the links between the circadian clock and prostate cancer, including melatonin, light exposure, and androgen dependence. In addition, the role of disrupted circadian rhythms in multiple types of cancer, with a specific focus on prostate cancer, will be explored in the context of molecular, epidemiologic, and clinical studies.

## Methods

A literature search was conducted to identify all literature on circadian rhythm and prostate cancer published between January 1, 2012, to September 1, 2022. Cochrane Library and PubMed were searched for relevant articles. The search strategy was performed using the keywords: prostate cancer and circadian rhythm, and prostate cancer and sleep-wake cycle. Relevant search results were imported into Mendeley Reference Manager. Additional studies were added to support the introduction and general discussion. Primary sources cited in articles included in the initial review were also included when relevant.

## The Circadian Clock

**Figure 1.**
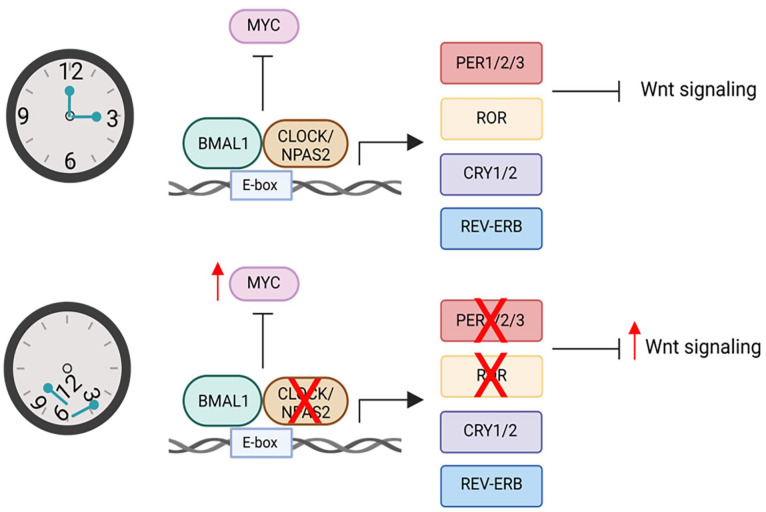
Potential roles of the circadian clock during prostate cancer tumorigenesis. BMAL1 indicates brain and muscle arnt-like protein 1; CLOCK, circadian locomotor output cycles kaput; CRY, cryptochrome; MYC, myelocytomatosis oncogene; NPAS2, neuronal PAS domain protein2; PER, negative regulators, period; ROR, related orphan receptor; REV-ERB or NR1D: nuclear receptor subfamily 1 group D.

The circadian clock is an evolutionarily conserved internal biological timekeeping system. Nearly every cell in the human body has a functional circadian clock that regulates critical cellular processes to maintain homeostasis.^3^ On a molecular level, circadian rhythms are generated by transcriptional-translational feedback loops. The core circadian clock genes include the positive regulators, brain and muscle arnt-like protein 1 (BMAL1) and circadian locomotor output cycles kaput (CLOCK), and the negative regulators, period (PER) and cryptochrome (CRY) (Figure 1). During the light phase, BMAL1 and CLOCK heterodimerize and promote the transcription of clock-controlled genes (CCGs) through consensus E-box motifs.^4^ However, BMAL1 and CLOCK also induce the expression of their negative regulators, the core clock genes PER and CRY. Throughout the light phase, PER and CRY levels accumulate and eventually repress the transcriptional effect of BMAL1 and CLOCK.^5^ This feedback loop occurs over a 24-hour period to coordinate gene expression patterns throughout the day.

Clocks within the body are entrained by external stimuli including light, temperature, and food to maintain robust rhythmicity over a 24-hour cycle.^6,7^ Disruption of these external stimuli through exposure to artificial light at night, erratic eating patterns, or rest-activity rhythms, often associated with night shift work, disrupt the circadian clock.^8,9^ Importantly, circadian clock disruption through night shift work has been named as a possible human carcinogen (group 2A carcinogen) by the International Agency for Research on Cancer (IARC).^10^ Thus, it is critical to understand the epidemiological and molecular consequences of circadian clock disruption on tumorigenesis. In the next sections, we will highlight the role of circadian clock disruption in tumorigenesis, with a specific focus on prostate cancer.

## Clinical and Molecular Evidence

Night shift work is associated with alterations to normal sleep-wake cycles and exposure to light at night which results in significant circadian rhythm disruption.^8,9^ Multiple studies have sought to define a correlation between night shift work and cancer incidence. Most studies have looked at the correlation between night shift work and breast cancer in nurses. Previous studies used follow-up surveys to track the rate of breast cancer incidence in mainly female nurses who did or did not participate in night shift work. Despite some contradictory conclusions, most these studies found that rotating night shift work increased the risk for developing breast cancer and that the risk often increased with a longer duration of shift work.^11,12^ In a more recent case-control study, the odds of developing breast cancer were twice as high in the women who worked night shift compared with those that did not.^13^ In addition to the studies on breast cancer, an association between night shift work and cancer has also been demonstrated for prostate,^14,15^ colorectal,^16-18^ lung,^19,20^ stomach,^21^ ovarian,^22^ and pancreatic^23^ cancers. A recent systematic review probed the effect of night shift work on cancer incidence from 57 separate studies including more than 8 million participants.^24^ In this study, there was no significant association between night shift work and the risk of breast, prostate, ovarian, pancreatic, colorectal, non-Hodgkin’s lymphoma, or stomach cancer.^24^ However, this study categorized participants based on ever or never having worked night shift, failing to take into account the duration of exposure to night shift work. Overall, the variability in data collection and categorization of night shift work warrants more thorough and comprehensive study to determine whether a strong association between shift work and cancer risk exists.

In a study that used data from The Cancer Genome Atlas (TCGA), 88.2% of clock genes were either upregulated or downregulated in at least one cancer type when compared with a matched normal sample.^25^ In addition to being differentially expressed in cancer, clock gene expression levels were clinically relevant in predicting patient survival and clinical stage.^25^ For example, CRY2 downregulation predicted worse patient survival in multiple cancer types.^25,26^ It has been suggested that disruption of the circadian clock promotes tumorigenesis through alteration of critical cellular processes that limit cancer progression. In support of this, clock genes were found to be associated with cell cycle control and activation of oncogenic pathways including phosphatidylinositol 3-kinase (PI3K)/protein kinase B alpha and Rat sarcoma/mitogen-activated protein kinase (MAPK).^25,26^ In addition, other studies have demonstrated an association between clock gene dysregulation and epithelial-mesenchymal transition (EMT),^25^ immune cell exhaustion,^26^ and cancer cell dissemination.^27^ These studies highlight the prognostic value of circadian clock gene expression in multiple cancer types.

Furthermore, there is evidence supporting the negative impact of circadian rhythm disruption on mitochondrial metabolism. Emerging evidence has described this interaction, through linking metabolism and circadian responses to transcriptional modifications.^28^ Akbari et al have hypothesized that depletion of adenosine triphosphate (ATP) drives normal cells toward cancerous transformation.^29^

Multiple genetic and environmental models of clock disruption demonstrate a significant increase in cancer progression.^30-34^ Accumulating evidence suggests an important connection between the circadian clock and regulation of the cell cycle.^30,35,36^ For example, PER2 mutation has been shown to promote tumorigenesis by deregulating critical cell cycle checkpoints and tumor suppressors.^35^ Similarly, CRY2 missense mutation suppresses p53 gene expression and enhances cell growth in *c-Myc* expressing fibroblasts.^36^ This introduces an intriguing connection between the circadian clock and the cell cycle through *c-Myc*, a CCG that acts as a key regulator of cell cycle progression and proliferation. In further support of this, BMAL1 expression levels correlate inversely with *c-Myc*^37,38^ and BMAL1 mutation has been shown to upregulate *c-Myc* expression.^39^ In contrast, Cry mutation decreased *c-Myc* expression.^39^ Intriguingly, BMAL1 mutation was shown to drive *Apc* loss of heterozygosity to upregulate *c-Myc* and drive colorectal cancer progression.^30^ Altogether, these data reveal that clock control of the cell cycle is critical for limiting cell growth and maintaining genome stability.

## Clock Disruption and Prostate Cancer

Although there is evidence that circadian clock disruption may promote the incidence and progression of various types of cancer, the role of clock disruption in the cause of prostate cancer is not yet well understood. There have been a few studies that have examined the role of circadian rhythm gene variants and prostate cancer risk.^40-42^ Initial studies attempted to define a correlation between single nucleotide polymorphisms (SNPs) in circadian genes and the risk of fatal prostate cancer. When comparing 3 separate cohorts of prostate cancer patients, no consistent association between circadian gene SNPs and prostate cancer fatality was found.^42^ However, this same study identified an association between CRY1 SNPs rs7297614 and rs1921126 and increased risk of fatal disease in 2 out of the 3 cohorts analyzed.^42^ These SNPs are predicted to influence splicing, suggesting a probable connection between the circadian clock and prostate carcinogenesis through proliferative signaling. In support of this, multiple studies have observed associations between clock genes known to regulate proliferative signaling, including CRY1, timeless circadian regulator, aryl hydrocarbon receptor nuclear translocator like (ARNTL), neuronal Per-Arnt-Sim domain protein2 (NPAS2), PER1/2/3, related orphan receptor α (RORα), CLOCK, and casein kinase 1 epsilon (CSNK1E), and the incidence or aggressiveness of prostate cancer^43-49^ (Table 1). The expression level of 9 core circadian clock genes has also been shown to correctly predict prostate cancer patient disease-free survival^50^ (Table 1). More broadly, individuals who suffer from sleep disorders or work night shift are at a higher risk of developing prostate cancer than those who do not.^23,51-53^ These studies demonstrate a potential role for the circadian clock in regulating prostate cancer initiation and progression.

**Table 1.** Clock gene functions in prostate cancer.

Citation	Clock gene and function
Markt et al^42^	CRY1 SNPs were nominally associated with prostate cancer fatality in 2 out of 3 cohorts studied.
Chu et al^43^	NPAS2 variation was associated with total prostate cancer risk in men treated with finasteride but not in the control group.
Wendeu-Foyet et al^44^	NPAS2 and PER1 variants significantly associated with all prostate cancer. RORA variants significantly associated with aggressive prostate cancer.
Wendeu-Foyet et al^45^	No significant association between SNPs and prostate cancer ARNTL, NPAS2, and RORA variants significantly associated with aggressive prostate cancer among night workers.
Park et al^46^	N-terminal domain of RORα1 downregulated genes involved in proliferation and tumor progression using RORα1-deficient mouse embryonic fibroblasts and prostate carcinoma tissues. RORα1 downregulation was associated with prostate cancer in patients.
Feng et al^47^	Using patient databases, CLOCK, PER, CRY2, NPAS2, RORA, and ARNTL were associated with anti-oncogenes and CSNK1D and CSNK1E were associated with oncogenes. Downregulation of PER1, PER2, and CRY2 and upregulation of CSNK1E were associated with higher risk of disease progression.
Gu et al^48^	NPAS2 and AANAT significantly associated with prostate cancer risk.
Yu et al^49^	NPAS2 SNPs were associated with a significantly higher risk of prostate cancer progression. NPAS2 downregulation was associated with more aggressive prostate cancer and poorer progression-free survival.
Yue et al^50^	Expression of CSNK1D, BTRC, CLOCK, CSNK1E, FBXL3, PRKAA2, DBP, NR1D2, and RORB was used to construct a risk score, and this score was found to be predict prostate cancer tumor status, disease, and progression-free survival and overall survival.
Li et al^54^	PER3 is downregulated in human prostate cancer samples, and this correlates with worse patient prognosis. Overexpression of PER3 in prostate cancer stem cell precursors suppressed their colony and sphere-forming abilities while knockdown of PER3 promoted their tumor-forming abilities.
Fedorova et al^55^	NETO2 mRNA expression did not correlate with clinicopathological features Knockdown of NETO2 in HCT116 cells downregulated cancer-associated pathways including PI3K/protein kinase B (AKT), JAK-STAT, Wnt, TGF-β, and MAPK pathways.

Abbreviations: AANAT, Arylalkylamine N-acetyltransferase; ARNTL, aryl hydrocarbon receptor nuclear translocator like; BTRC, beta-transducin repeat containing E3 ubiquitin protein ligase; CLOCK, circadian locomotor output cycles kaput; CRY, cryptochrome; DBP, D-box binding PAR (proline- and acid-rich) bZIP (Basic leucine zipper) transcription factor; JAK, Janus kinase; MAPK, mitogen-activated protein kinase; NPAS, neuronal PAS domain protein2; PER, negative regulators, period; PI3K, phosphatidylinositol 3-kinase; RORA, retinoic acid receptor-related orphan receptor alpha; ROR, related orphan receptor; SNPs, single nucleotide polymorphisms; STAT, signal transducer and activator of transcription; TGF, transforming growth factor.

NPAS2, a core component of the circadian clock, has been identified as a potential link between the circadian clock and prostate carcinogenesis. NPAS2, along with other transcription factors (CLOCK, ARNTL/BMAL1, or ARNTL2/BMAL2) form the positive limb of the feedback loop and activate the transcription of core clock genes and CCGs^56^ (Figure 1). NPAS2 is homologous to CLOCK and plays a role in the DNA damage response through inhibition of cell cycle and DNA repair genes.^57^ Two meta-analyses of data, including from the Genetic Associations and Mechanisms in Oncology (GAME-ON) network, using genome-wide association studies have also showed that NPAS2 is one of the genes with the most significant contribution in the association between the circadian pathway and the risk of prostate cancer.^48,58^ Similarly, data from the Epidemiological Study of Prostate Cancer (EPICAP) study were used to demonstrate an association between NPAS2 and aggressive prostate cancer, especially in those who performed night shift work, with risk positively correlating to duration of night shift work.^44,45^ In addition, associations between prostate cancer risk and NPAS2 were found on the SNP level where rs6542993 predicts prostate cancer risk for both localized and metastatic diseases.^49^ *NPAS2*rs6542993 was significantly associated with biochemical recurrence of prostate cancer (*P* *=* *.039*) and also found to be associated with an increased risk of progressive disease, after adjusting for known clinicopathological variables that are associated with advanced prostate cancer, confirming *NPAS2* rs6542993 as a biomarker for prostate cancer progression.^49^

Although multiple studies have found an association between NPAS2 and prostate cancer risk, few have identified a mechanism regulating this association. However, 2 key studies provide clues for a potential mechanism. Chu et al used data from the prostate cancer prevention trial (PCPT), a randomized placebo-controlled clinical trial for the efficacy of finasteride, to define the relationship between circadian gene variants and prostate cancer risk as well as the effect of testosterone on this relationship. In the group treated with finasteride, they demonstrated an association between NPAS2 and prostate cancer risk that was lacking in the placebo group.^43^ This suggests that NPAS2 variation may increase risk of prostate cancer through androgen receptor–dependent signaling.

Other interesting links between the circadian clock and prostate cancer are through retinoic acid-RORα1, PER3, and their control of Wnt signaling. Related orphan receptor α1 is a transcription factor known to be involved in regulating circadian rhythms,^59^ metabolism,^60^ and immunity.^61^ Downregulation of RORα1 hyperactivates proliferative genes including Wnt target genes.^46^ Interestingly, knockdown of PER3 in prostate cancer stem cells significantly promotes tumorigenic properties and similarly activates Wnt signaling^54^ (Table 1). Both RORα1 and PER3 are downregulated in prostate cancer specimens and inversely correlated to Wnt signaling.^46,54^ In the intestine, the circadian clock regulates the rhythmic secretion of Wnt ligands and intestinal stem cells are highly responsive to Wnt activation.^62-64^ Thus, it is plausible that a similar mechanism involving circadian regulation of Wnt signaling may also be operative in prostate cells, contributing to the regulation of stemness and tumorigenic properties of prostate cancer cells. In addition, increased expression of neuropilin and tolloid-like 2 (NETO2), a protein involved in regulation of kainite receptor function, was found to dampen circadian rhythms in prostate cancer leading to upregulation of Wnt signaling^55^ (Table 1). Overall, these studies demonstrate that prostate cancer may be mediated by circadian rhythms through regulation of Wnt signaling which provides a potential therapeutic target for future studies.

Finally, there is evidence supporting the importance of sleep hygiene as a determining factor of mitochondria health. There is an interplay between the sleep cycle and mitochondrial interactome disturbances that are suspected to influence the pathogenesis of cancer, among other diseases. This interplay can be categorized into 3 main categories of redox regulation, bioenergetics regulation, and temperature regulation.^65^

## Melatonin and Circadian Rhythm

Ongoing studies investigate the role of N-acetyl-5-methoxytryptamine (melatonin) and its role in prostate cancer. It has been well-established that melatonin is a principal regulator of the circadian rhythm.^66^ Melatonin also seems to play a role in numerous solid tumors, and investigations in terms of its role as a potential molecule that impedes the spread of cancer are ongoing.

In prostate cancer, as described in the prostate cancer lifestyles (CAPLIFE) study, the saliva of patients was collected to measure melatonin levels of patients and controls. Melatonin levels were used as a proxy for circadian rhythm disruption. It was found that patients with prostate cancer had lower levels of melatonin, regardless of urinary symptomatology, tumor aggressiveness and extension, and chronotype. In addition, the maximum melatonin peak was lower in prostate cancer patients when compared with controls.^67^ When melatonin levels were studied in a case-cohort study of 928 Icelandic men, a similar association was found in morning urine samples, as men with lower levels of first morning-void urinary 6-sulfatoxymelatonin were at increased risk for advanced or lethal prostate cancer.^68^ In a systematic review and meta-analysis, men with lower melatonin levels had an increased risk of prostate cancer, and low melatonin levels increased the incidence of advanced prostate cancer.^69^ These studies suggest a potential link between melatonin and prostate cancer incidence and/or progression. Several other studies have examined the synergistic use of melatonin with androgen deprivation therapy (ADT) in patients with prostate cancer^70-72^ (Table 2).

**Table 2.** Use of melatonin and ADT in prostate cancer.

Li et al^54^	In castrated mice, melatonin treatment was associated with a decrease in lymph node carcinoma of the prostate tumor incidence and growth rate.
Fedorova et al^55^	Fourteen metastatic prostate cancer patients received both triptorelin, a GnRH agonist, and melatonin, and 8 of 14 (57%) patients had a greater than 50% decrease in serum PSA level.
Zhou et al^69^	In prostate cancer patients who received combined hormone and radiation treatment, in the group of patient with poor prognosis, patient who received melatonin had on average a 13-month longer survival rate when compared with control.

Abbreviations: ADT, androgen deprivation therapy; PSA, prostate-specific antigen.

Whether or not melatonin can play a role in transcriptional activity related to prostate cancer progression remains under investigation. There are a number of integrin receptors and their corresponding ligands that have been investigated to better understand how their expression patterns may be related to prostate cancer and its progression. One such integrin is integrin α2β1, which is highly expressed in prostate cancer. Evidence suggests that its activation/phosphorylation has been implicated in prostate cancer progression.^73^ Expanding on this, Tai et al examined inhibition of this integrin in a study of 2 human osteoblastic cell lines, which were stimulated with melatonin. In this study, melatonin induced the suppression of osteoblastic prostate cancer cell motility by playing a role in inhibiting the integrin α2β1. This inhibition took place by way of inhibiting focal adhesion kinase, c-Src, and nuclear factor-κB transcriptional activity via the melatonin MT_1_ receptor.^74^ Suppression of these cell’s motility translated into decreased migration and invasive ability of these cell lines. Skeletal metastases are not uncommon in advanced prostate cancer, and the role of integrin α2β1 is crucial in this process.^75^ Perhaps, this link provides an explanation of the role of melatonin in suppressing the proliferation of skeletal metastases in patients with advanced prostate cancer, and potentially even in slowing the rate of progression of disease. Further studies on this topic are warranted.

Another area under investigation when it comes to prostate cancer and melatonin is more specifically the role of melatonin in lipid metabolism. Prostate cancer is known to be a lipid-rich tumor,^76^ and the role of dietary lipids has been studied in its development and progression. The circadian rhythm is well-known to regulate lipid homeostasis, and disruptions of this temporal regulation have been shown to lead to impaired lipid absorption and ultimately, to metabolic disorders, and even tumor development.^77-79^ Although increased lipogenesis is initially androgen-responsive, it persists or re-emerges in castrate-resistant prostate cancer, suggesting that lipid metabolism outside of the setting of androgen sensitivity plays a fundamental role in the progression of prostate cancer.^80^ In addition, in 1 study using mouse-derived prostate cells to generate a prostate cancer model, melatonin was found to promote the expression of the Carboxylesterase 1 (CES1) gene, which is lipid metabolism-related. In prostate cancer tissues compared with normal prostate tissue, the expression of CES1 was downregulated and high levels of CES1 expression were negatively correlated with tumor stage, metastasis, and Gleason score. In this same study, melatonin upregulated CES1 expression, which decreased lipid accumulation and cell activity by prostate cancer cells, as well as inhibited castrate-resistant prostate cancer progression, and reversed enzalutamide progression.^69^

## Light and Circadian Rhythm

A discussion of the role of melatonin and prostate cancer is incomplete without a discussion about light. As we know, melatonin is released under the control of the circadian rhythm and exposure to light. Regular exposure of humans to artificial light at night has been linked to an increased risk of prostate cancer in men with normal sight.^81^ The hypothesized mechanisms for this are nocturnal melatonin synthesis suppression, circadian time structure desynchronization, and sleep/wake cycle disruption with sleep deprivation. Night time use of personal electronic devices has contributed to this. The combination of vitamin D suppression in the modern day in addition to suppressed melatonin synthesis is thought to create a manmade light environment with potentially toxic consequences.

Another study used human prostate cancer xenografts in rats to test night time melatonin levels after exposure to white light through blue-tinted versus clear cages. Daytime blue light stimulated about a 6-fold increase in the peak night time melatonin level when compared with the normal peak night time melatonin level. The mechanism for this is unknown. It was also found that there was amplification of night time melatonin levels in the group exposed through blue-tinted cages, which reduced human prostate cancer metabolic, signaling, and proliferative activities.^82^ Blue light is emitted by many outdoor lights, phones, tablets, and other personal electronic devices in the home including TVs and laptops. In a Spanish study, exposure to outdoor light at night in the blue light spectrum was associated with a higher odds of prostate cancer (odds ratio [OR] = 2.05; 95% confidence interval [CI] = 1.38-3.03).^83^ Although new and more conservative prostate cancer screening guidelines by way of prostate-specific antigen (PSA) led to declining prostate cancer incidence rates in the late 2000s and 2010s. Between 2014 and 2018, incidence rates for advanced-stage prostate cancer rose by 4% to 6% each year.^84^ It is difficult to ascertain what role personal electronic devices and the manmade light environment may have in this process, and again, further investigation is warranted.

Altered light exposure dysregulates circadian rhythms, and it is thought that the biological effects of circadian rhythm disruption may affect androgen expression, androgen receptors, cyclin D1, cell proliferation, apoptosis, and repair.^85^ In 1 case study obtained from the GLOBOCAN 2002 database on light-at-night and prostate cancer, the increase in light-at-night from minimal average exposure to average exposure corresponded to an increase in prostate cancer age-standardized rate by 30.5%. When light-at-night was further increased to the maximum average exposure, this corresponded to an increase in the prostate cancer age-standardized rate by 80.2%.^86^ Surely, there may have been other confounding variables in this study that would need to be tightly correlated with light-at-night exposure to have made a significant difference. Interestingly, in another study, there was a significant positive association between population exposure to light-at-night and incidence rates of prostate cancer, but no such association with lung cancer or colon cancer.^87^ Again, this suggests a possible role of hormone mediation, as prostate cancer typically originates as a hormone-sensitive cancer, whereas this is not the primary pathophysiology of lung cancer and colon cancer. Perhaps in prostate cancer, melatonin and testosterone both play independent and dependent roles in contributing to the development and progression of disease, and both hormones are dysregulated by circadian rhythm disruption.

## Shift Work and Prostate Cancer

Some studies have suggested a positive association between the presence of shift work and the risk of prostate cancer. In a meta-analysis conducted in 2018 including a total of 10 715 prostate cancer patients, shift work was significantly associated with an increased risk of prostate cancer.^88^ Exposure to light-at-night leading to circadian rhythm disruption and endocrine disruption in humans has been thought to have implications on metabolic disorders, as well as on endocrine-related cancers which include prostate cancer.^89^ One study assessed the incidence of cancer among commercial airline pilots and found that airline pilots had higher rates of prostate cancer.^90^ Another study examined night shift work with relation to prostate cancer and found that individuals who worked for at least 1 year in night shift work again had higher prostate cancer risk.^18^ This study also found an association between night shift work and prostate cancer tumors with a worse prognosis. In the CAPLIFE study, when looking at the effect of shift work, night shift work was associated with prostate cancer with an adjusted odds ratio (aOR) = 1.47 (95% CI = 1.02-2.11), especially for rotating night shifts, aOR = 1.73 (95% CI = 1.09-2.75).^91^ An increased risk for evening chronotypes was also established.

On the contrary, in a population-based cohort study in Sweden, there was no increased risk of prostate cancer among shift workers compared with the general population of Swedish men.^92^ The definition of shift work in this study did not have to include night shift work, although night shift work is associated with more disruption in the circadian rhythm when compared with day shift work. A twin study, also out of Sweden, with data extracted from the Swedish Twin Registry and the Swedish Cancer Registry, found that there was no association between ever night work and prostate cancer.^93^ Likewise, a Finnish twin study also found no significant association between shift work and prostate cancer risk.^94^ To our knowledge, no studies have examined light patterns and circadian rhythm as they relate to geographic location, and correlated this to patterns in cancer epidemiology. This would provide for an interesting investigation that may lead to new insights. Furthermore, when it comes to defining night shift work, there is room for misclassification. Night shift work may be self-reported or based on job title. Within this group of night shift workers, there may also be rotating shift workers who alternate between day shift and night shift. There are scarce data looking at the risk of prostate cancer and circadian rhythm disruption between these subgroups, although differences may exist.

Overall, the studies on night shift work and prostate cancer do show mixed results.^53,95^ In a systematic review reviewing the literature from 2012 to 2017, it was concluded that evidence of an association between night shift work and prostate cancer is inconclusive.^95^ In a meta-analysis ultimately including 9 relevant studies, interestingly, rotating night shift work was associated with a significantly increased risk of prostate cancer, while fixed night shift work was not.^96^ In another study, prostate cancer risk was found to decrease following the cessation of night shift work, suggesting that the pattern of time-related decrease in risk may exist. The pattern has also been observed in breast cancer.^97^ Several studies have alternatively suggested that night shift work is not associated with the risk of developing prostate cancer. When looking at risk of fatal prostate cancer, work schedule and insomnia frequency were not significantly associated.^98^ The CAPLIFE study did not find a clear trend between exposure time in night work or inadequate hours of sleep and prostate cancer risk.^91^ In the PROtEuS study, a population-based case-control study from Canada, including 1904 prostate cancer cases, no association was found between overall prostate cancer and night shift work metrics, including ever exposure, duration, intensity, cumulative exposure, rotating shifts, and early-morning shifts.^14^ In the EPICAP study, a French population-based case-control study including 818 prostate cancer cases, night work was not associated with prostate cancer, regardless of how aggressive the prostate cancer was. However, at least 20 years of permanent night work was associated with aggressive prostate cancer, and this was more so found in men who worked shift lengths >10 hours, or ⩾6 consecutive nights.^15^ Another major systematic review on the topic reported similar findings, with results not supporting the hypothesis that rotating shift or night work schedules are associated with a higher risk of prostate cancer.^99^

Overall, when looking at the observational studies that have tried to evaluate the association between night shift work and prostate cancer, it is clear that the findings are mixed. Perhaps, the great heterogeneity in methods and participants is a major uncontrolled factor in these studies. Factors such as work schedule variation, geographic location, age, and publication bias may all play roles in the heterogeneity of findings.

## Chronotherapy

Chronotherapy refers to the coordination of medical treatment with biological rhythms to maximize therapeutic drug efficacy and minimize side effects.^100^ The human circadian clock has traditionally served as the basis for which chronochemotherapy regimens were designed.^101^ Although this method has been beneficial in certain medical realms, when it comes to clinical trials using chronochemotherapy, this method did not show improved pharmacologic efficacy, and even showed worse clinical outcomes in subsets of patients when compared with conventionally timed therapies.^102^ It is important to note that the models that have simulated the circadian clock have limitations, as they analyze only the core clock genes and those in the secondary consolidating loop, which is a gross simplification of the hundreds to thousands of genes presumed to play a role in the human circadian rhythm.^101^

Looking at trials in human malignancies, there has been work done in both gynecologic cancers and colorectal cancer examining chronochemotherapy. In advanced ovarian cancer, a clinical trial of 31 patients showed differences in the group receiving adriamycin in the evening and cisplatin in the morning, as opposed to adriamycin in the morning and cisplatin in the evening. The group receiving adriamycin in the evening required more dose attenuation and treatment delays as opposed to the other group, and treatment complications were still about 2 times more common as in the group receiving adriamycin in the morning and cisplatin in the evening.^103^ A larger phase 3 trial with 342 total patients (of which 169 patients were in the control arm) showed that in patients with advanced or recurrent endometrial carcinoma, there was no significant benefit in terms of response rate, PFS or OS, or toxicity profile in patients who received chronochemotherapy as opposed to the standard treatment timing.^103^ The chronotherapy pattern in this study matched that of the aforementioned study of ovarian cancer patients. Currently, there are no guidelines for the use of chronochemotherapy in the United States, and no clinical trials to our knowledge that have looked at chronochemotherapy in the treatment of prostate cancer.

Interestingly, there have been multiple reports on the use of chronoradiation therapy in prostate cancer patients, as well as how that relates to micturition. In 1 study conducted in men with localized prostate cancer, there was a significant improvement in lower urinary tract symptoms and quality of life with morning proton beam therapy (PBT) when compared with noon time or afternoon PBT.^104^ In another study looking at differences in toxicity and outcomes in patients with prostate cancer undergoing daytime vs evening radiation, patients with T2b or higher T-stage had significantly poorer biochemical failure-free survival when treated in the evening.^105^ A literature review looking at 9 studies regarding the timing of radiotherapy and its potential effect on clinical outcomes showed that inconsistencies in the literature exist on this topic, and that further investigation in preclinical experiments is necessary to better understand the effect of circadian rhythms and radiotherapy on cell cycle progression.^106^

## Androgen-Deprivation Therapy and Insomnia

It is estimated that 25% to 40% of prostate cancer patients experience insomnia, and ADT, radiation therapy, and prostatectomy are all believed to play a role.^107,108^ There is certainly a lack of trials examining sleep disorders in prostate cancer patients, and thus, a dearth of prospective data on the topic. From reviewing the limited retrospective literature, as Sparasci et al most recently completed, 14 of 16 trials demonstrated development of sleep disorders or changes in sleep quality in this patient population.^107^ Hot flashes and nocturia are prominent factors that play a role in contributing to insomnia in men with prostate cancer undergoing treatment. Patients on ADT report greater hot flash incidence and more severe and clinically significant sleep disturbances when measured on the Insomnia Severity Index.^109^ In 1 small randomized controlled clinical trial (total n = 42, 21 patients in melatonin arm and 21 in placebo group) investigating the role of melatonin on sleep and mood in prostate cancer patients on ADT, melatonin significantly improved sleep, measured by the Pittsburgh sleep quality index.^110^ Additional studies investigating the role and mechanisms of ADT on the circadian rhythm and sleep are needed.

## Androgen Independence

Gaining a deeper understanding of the role of circadian rhythm in its potential role, if any, in contributing to prostate cancer progression and/or androgen independence, is a question that remains. Finally, little is known about the mechanism by which prostate cancer cells hijack the expected response to androgen receptor suppression and become androgen-independent. Often times, they grow to resemble a neuroendocrine-like disease state. The idea that prostate cancer cells can be driven toward androgen independence via the pathway of drug-induced epigenomic plasticity which reprograms circadian rhythm regulation has recently been explored in a phase 2 clinical trial by Linder et al. This study demonstrated that in tissues of patients with high-risk prostate cancer treated with 3 months of enzalutamide monotherapy, there was reprogramming in pioneer factor forkhead box protein A1 *(FOXA1)*, which ultimately led to these *FOXA1* sites being enriched for the circadian clock component *BMAL1*.^111^ BMAL1 is of significance as it forms a heterodimer with CLOCK, and is a transcriptional activator which forms a core component of the circadian clock.^112^ In this study mentioned above, high *BMAL1* levels after enzalutamide treatment were associated with poor clinical outcomes. *BMAL1* levels were also exclusively found to be upregulated in nonresponders, defined as patients having biochemical recurrence in less than or equal to 6 months after surgery. This study suggests that the circadian regulator *BMAL1* may be a novel candidate therapeutic target.^111^

## Conclusion

The potential role of circadian clock dysregulation in the development and progression of prostate cancer has been relatively unexplored. Limited molecular, epidemiologic, and clinical studies have addressed this topic. The influence of melatonin, light, night shift work, chronotherapy, and androgen independence may all play roles in the link between the circadian clock and prostate cancer. Night shift work results in significant circadian rhythm disruption; however, observational studies evaluating the role of night shift work on prostate cancer have provided for mixed and overall inconclusive results. There is much room for further studies to broaden our understanding on this topic, and more generally, on the topic of how dysregulated circadian rhythms contribute to tumorigenesis. Gaining a deeper understanding of the role of the circadian clock in its potential role, if any, in contributing to prostate cancer progression and/or androgen independence, is a question that remains.

The circadian clock regulates critical cellular functions to maintain homeostasis. While the role of the circadian clock in prostate cancer tumorigenesis is not fully understood, circadian clock genes have been linked to prostate cancer risk and aggressiveness. For example, NPAS2, homologous to CLOCK and regulating the cell cycle, is associated with prostate cancer risk,^43,48^ aggressiveness,^44,45^ and progression.^49^ NPAS2:BMAL1 heterodimers have been shown to regulate the expression of the proto-oncogene and Wnt target c-MYC, highlighting a potential mechanism of NPAS2 in tumorigenesis.^35^ In addition, other circadian clock genes including RORα1 and PER3 have associated with prostate cancer. RORα1 and PER3 have been found to be downregulated in prostate cancer samples and inversely correlated to Wnt signaling.^46,54^ Overall, circadian regulation of Wnt signaling may be involved in prostate cancer tumorigenesis.
